# A fetus of partial urorectal septum malformation sequence characterized by complete septate uterus: A case report

**DOI:** 10.1097/MD.0000000000033448

**Published:** 2023-03-31

**Authors:** Jingfang Zhai, Shenghan Cao, Xuezhen Wang, Ying Liu, Bei Zhang

**Affiliations:** a Graduate School of Bengbu Medical College, Bengbu, Anhui, China; b Department of Prenatal Diagnosis Medical Center, Xuzhou Central Hospital, Xuzhou Clinical School of Xuzhou Medical University, Xuzhou, Jiangsu, China; c Key Laboratory of Brain Diseases Bioinformation of Xuzhou Medical University, Xuzhou, Jiangsu, China.

**Keywords:** abdominal cystic mass, autopsy, prenatal diagnosis, urorectal septum malformation sequence

## Abstract

Urorectal septum malformation sequence (URSMS) is an extremely uncommon anomaly characterized by imperforate anus accompanied by multiple genitourinary malformations. Here, we report a case of URSMS identified by the autopsy and classified into partial URSMS. Prenatal diagnosis is challenging for clinicians due to the difficulty of early identification of URSMS and the relative lack of specific features in ultrasound. We intend to share our experiences.

**Patient concerns:**

One fetus was indicated abdominal cystic structure, abdominal effusion and right renal pelvis separation (7 mm) by ultrasound at 28 + 1 week’s gestation. After the pregnancy was terminated, the fetal tissues were performed to be tested by autopsy, copy number variation sequencing and whole exon sequencing.

**Diagnoses:**

Based on the clinical characteristics, ultrasound, autopsy, and genetic test findings, the fetus was diagnosed with URSMS.

**Interventions:**

After genetic counseling, the couple opted to terminate her pregnancy.

**Outcomes:**

The copy number variation results of the fetus showed a 0.48-MB duplication fragment of uncertain significance on chromosome 8p23.3, while the whole-exome sequencing revealed a SAL-LIKE 1 gene mutation. The autopsy of the fetus showed imperforate anusa, the abdominal cyst was further confirmed with complete septate uterus and the lower urethra and vagina converge formed a lumen.

**Lessons:**

Individuals with URSMS during the fetal period might be misdiagnosed due to atypical features of URSMS. Once structural abnormalities especially cystic mass of the futuses in the lower abdomen, URSMS should be considered.

## 1. Introduction

The urorectal septum malformation sequence (URSMS) is a rare abdominal structural malformation with an incidence rate of 1/250,000 to 1/50,000 of newborn babies, firstly reported in 1987.^[1]^ Its clinical features are mainly characterized by imperforate anus accompanied by multiple genitourinary malformations. The pathogenesis is still unclear, however, the pathogenetic mechanism is commonly accepted that URSMS is resulted from the urorectal septum failing to separate or fuse during in the early stage of embryonic development. In clinical work, the fetuses with URSMS are commonly missed due to a lack of direct diagnosis sign of URSMS in prenatal ultrasound, and most of them are confirmed by autopsy after fetal death. In this report, a fetus was diagnosed with URSMS and terminated, and we intend to share our experiences during the process of the fetus with URSMS during the fetal period.

## 2. Case presentation

**Figure 1.**
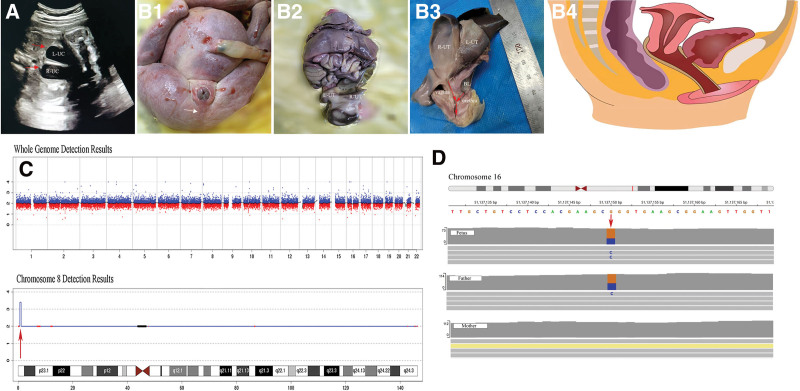
(A) Ultrasound image at 28 + 1 weeks of gestation showed a 5.5 × 3.1 cm abdominal cystic structure of the fetus and was confirmed as complete septate uterus confirmed by the autopsy. (B) Autopsy of the fetus: (B1) the fetal appearance of the abdomen and vulva with imperforate anus; (B2) the fetal appearance of posterior view of abdominal anatomy; (B3) the lower urethra and vagina converge to form a lumen (arrowhead); (B4) diagram map of the autopsy. (C) The fetal CNV-Seq result showed a 0.48-Mb duplication on 8p23.3p23.3 (580000_1060000). (D) WES indicated SALL1 missense mutation on chr16: 51137150-51137150: NM_002968.3: c.3937G > C (p.R1313G) on exon3. Annotation: BL = bladder, CNV-seq = copy number variation sequencing, L-UC = left uterus cavity; R-UT = right uterus cavity, SALL1 = SAL-like 1, WES = whole-exome sequencing.

A 28-year-old primipara was referred to prenatal diagnosis medical center of Xuzhou Central Hospital due to abnormal ultrasound results without any histories of adverse pregnancy, drug usage and non-consanguineous marriage. Prenatal ultrasound in local hospital demonstrated single umbilical artery, seroperitoneum, rectal, partial colon dilatation (considering anal atresia?), mild hydronephrosis of right kidney presented at 27 + 6-week gestation (WG). At 28 + 1 WG, a 5.5 × 3.1 cm abdominal cystic structure, abdominal effusion, a 7 mm separation of the right renal pelvis was revealed in our ultrasound diagnosis center (Fig. 1A). The couple chose to terminate the pregnancy after multiple genetic counseling and the fetus tissues were performed to be tested by autopsy, copy number variation sequencing and whole exon sequencing (WES) after fully written consent. The autopsy of the fetus showed imperforate anusa (Fig. 1B1), 2100-g fetus, the abdominal cyst confirmed with complete septate uterus (Fig. 1B2), the lower urethra and vagina converge to form a lumen (Fig. 1B3), and the diagram map of abdominal anatomy (Fig. 1B4). The fetal copy number variation sequencing result showed a 0.48-Mb duplication fragment on 8p23.3p23.3 (580000_1060000) variants of unknown significance (Fig. 1C). The result of WES indicated that no pathogenic SNV and InDel variants related to the phenotype of this case, however, a genetic SAL-like 1 (SALL1) missense mutation on chr16: 51,137,150 to 51,137,150: NM_002968.3: c.3937G > C (p.R1313G) on exon3 (Fig. 1D) was revealed to be possibly related to the phenotype of this case derived from the fetus father. This study was approved by Xuzhou Central Hospital Ethics Committee. Informed consent was obtained from the fetus parents for the publication of our case.

## 3. Discussion

URSMS is commonly caused between 6th and 7th WG by the failure of urorectal septum to separate the cloacal cavity or to fuse with the cloacal membrane.^[2,3]^ The patients with URSMS commonly present with urorectal septum dysplasia and urogenital abnormalities, among which septate uterus is caused by obstructed absorption of mediastinum after fusion of bilateral accessory mesonephros ducts from 10th to 12th WG and the crista-shaped septum remains in the uterine cavity,^[4]^ while congenital anal atresia results from the obstruction of the migration from the urogenital diaphragm to the cloaca from the 4th to 8th weeks of the embryo. Considering our fetus, it had multiple malformations including septate uterus, anal atresia and fusion changes of vagina and urethra, hence the process of the fetus development was externally affected from 4th to 12th WG. Based on persistent cloacal and perineal openings, URSMS are classified as complete and partial 1.^[2]^ Our case with URSMS is classified as a partial urorectal septum sequence confirmed by autopsy, who was characterized by the presence of a single perineal outflow as an opening for the common channel of both urogenital tract and genitourinary tract. The infants with partial URSMS have been reported with relatively good prognoses without severe cardiopulmonary and renal dysfunction, however, the survivors still need multiple genitourinary and intestinal reconstruction operations in the neonatal period.^[2]^ Therefore, the prompt and correct diagnosis of URSMS during the pregnancy are particularly essential for the couple whether to continue their pregnancy. Perhaps we failed to diagnose URSMS correctly in time and the couple insisted on termination of pregnancy, finally after receiving sufficient genetic counseling, the couple finally chose to terminate the pregnancy.

Although prenatal ultrasound, as a helpful tool, has no direct diagnostic significance for URSMS, it has a certain value of guideline for the screening of characteristic URSMS. The abdominal cystic mass was recognized as the first symptom of URSMS.^[5]^ Achiron et al^[6]^ reported that prenatal diagnosis of URSMS was feasible using ultrasound criteria of enlarged bowel with echogenic foci and ambiguous genitalia. Moreover, Anal atresia occurred in 24 of the 25 cases reported by Wheeler et al^[2]^ In our case, prenatal ultrasound revealed multiple abnormalities in the fetus, anal atresia is the most prominent feature in particular. Prenatal clinicians should consider the possible diagnosis of URSMS once encountering an abdominal cystic mass with imperforate anus with genitourinary abnormalities and renal dysplasia. Ambiguous external genitalia are often hard to recognize the sex of the patients. Hence, the comprehensive and initial evaluation of these patients in clinic should include chromosomal analysis, renal ultrasound, and imaging of the internal cloaca, if tethering is suspected, spinal cord radiographs or magnetic resonance imaging.^[7]^ The characteristics of cloacal exstrophy variants are similar to those of URSMS, which comprise a wide range of disorders with 4 primary features: Omphalocele, bladder exstrophy, an imperforate anus, and spina bifida.^[8]^ So autopsy is helpful for us to identify them. In addition, URSMS have similar abnormal overlap with the vertebral defects, anal atresia, tracheo-esophageal fistula, renal defects, and radial dysplasia (VATER). Chien et al^[9]^ found that the patients with URSMS had urinary tract defects, anal defects, higher incidence of atresia, and genital abnormalities compared with VATER. Commonly, the usual vertebrae in VATER mostly occurs in thoracolumbar hemivertebra, while sacral hypoplasia is present in URSMS.

In addition, the genetic mechanism of URSMS is not clear up to now, which might be related to genetic diseases as a autosomal recessive inheritance or an X-linked mode.^[10,11]^ The genes of homeobox A13 and homeobox D13 related with sonic hedgehog pathway may be associated with URSMS.^[12]^ Nakata et al^[13]^ proposed candidate genes such as fibroblast growth factor 10, wingless-type mmtv integration site family, member 5a should be related with URSMS. While, a SALL1 gene mutation was identified by WES in our case. SALL1, which, as a strong transcriptional repressor, is expressed in a variety of tissues during embryonic development such cellular blastula layer.^[14]^ A series of pathways including sonic hedgehog, What are involved in the activation of SALL1 expression in different tissues. Hence, the SALL1 multation might lead to the malformations of different tissues.^[15]^ Si Dong et al^[16]^ in 2003 verified the effect of losing of the spalt gene (the homolog of human SALL1 gene) in the drosophila melanogaster, which yielded morphologic defects in the testes, genitalia, and antennareported defects similar to those in humans with Townes-Brocks syndrome. However, there are no reports of SALL1 gene variation causing URSMS in human beings to this day.

In conclusion, URSMS is a complex congenital abnormal syndrome, which is difficult to diagnose prenatally, and the corresponding clear diagnosis is vital important for the pregnant parents to choose whether to continue pregnancy or not, to receive the following genetic consultation and the related treatment. Once fetal abdominal cystic mass of unknown nature is detected by prenatal ultrasound especially accompanied by multiple urinary and genital abnormalities and anal atresia, URSMS should be highly suspected, further confirmed, and evaluated the possible prognosis.

## Acknowledgments

We would like to thank for the couple’s participation and the staff’s cooperation of the department of Prenatal Diagnosis Medical Center and Ultrasound of Xuzhou Central Hospital.

## Author contributions

**Conceptualization:** Jingfang Zhai, Bei Zhang.

**Data curation:** Jingfang Zhai, Ying Liu.

**Funding acquisition:** Jingfang Zhai.

**Supervision:** Jingfang Zhai, Bei Zhang.

**Writing – original draft:** Shenghan Cao, Xuezhen Wang.

**Writing – review & editing:** Jingfang Zhai, Bei Zhang.
